# Microwave Scissors-Based Sutureless Laparoscopic Partial Nephrectomy Versus Conventional Open Partial Nephrectomy in a Porcine Model: Usefulness and Complications

**DOI:** 10.1245/s10434-024-15548-7

**Published:** 2024-06-08

**Authors:** Ha Ngoc Nguyen, Atsushi Yamada, Shigeyuki Naka, Koichiro Murakami, Soichiro Tani, Tohru Tani

**Affiliations:** 1 Department of Advanced Medical Research and Development, Shiga University of Medical Science, Shiga, Japan; 2 Department of Urology, Faculty of Medicine, University of Medicine and Pharmacy at Ho Chi Minh City, Ho Chi Minh City, Vietnam; 3 Medical Innovation Research Center, Shiga University of Medical Science, Shiga, Japan; 4 Department of Surgery, Hino Memorial Hospital, Shiga, Japan; 5 Department of Surgery, Shiga University of Medical Science, Shiga, Japan; 6 Department of Surgery, Nagaokakyo Hospital, Kyoto, Japan

**Keywords:** Laparoscopic partial nephrectomy, Renorrhaphy, Microwaves, Renal function, Renal ischemia

## Abstract

**Background:**

This study aimed to compare the benefits and safety of microwave scissors-based sutureless laparoscopic partial nephrectomy (MSLPN) with those of conventional open partial nephrectomy (cOPN).

**Methods:**

Each kidney in nine pigs underwent MSLPN using microwave scissors (MWS) via transperitoneal laparoscopy or cOPN via retroperitoneal open laparotomy. The kidney’s lower and upper poles were resected under temporary hilar-clamping. The renal calyces exposed during renal resections were sealed and transected using MWS in MSLPN and were sutured in cOPN. For MWS, the generator’s power output was 60 W. Data on procedure time (PT), ischemic time (IT), blood loss (BL), normal nephron loss (NNL), and extravasation during retrograde pyelogram were compared between the two techniques.

**Results:**

The authors successfully performed 22 MSLPNs and 10 cOPNs. Compared with cOPN, MSLPN was associated with significantly lower PT (median, 9.2 vs 13.0 min;* p* = 0.026), IT (median, 5.9 vs 9.0 min; *p* < 0.001), BL (median, 14.4 vs 38.3 mL; *p* = 0.043), and NNL (median, 7.6 vs 9.4 mm; *p* = 0.004). However, the extravasation rate was higher in the MSLPN group than in the cOPN group (54.5 % [*n* = 12] vs 30.0 % [*n* = 3]), albeit without a significant difference (*p* = 0.265). Pelvic stenosis occurred in one MSLPN procedure that involved deep lower pole resection near the kidney hilum.

**Conclusions:**

The study data show that MSLPN can improve intraoperative outcomes while reducing technical demands for selected patients with non-hilar-localized renal tumors. However, renal calyces, if violated, should be additionally sutured to prevent urine leakage.

Partial nephrectomy (PN), which preserves an affected kidney’s tissue and the corresponding renal function (RF)^1^ while achieving oncologic control^2^ similar to that of radical nephrectomy, is the standard treatment for T1 renal cell carcinoma. The PN procedure can be performed with open (OPN), laparoscopic (LPN), or robotic (RPN) surgery, conventionally involving hilar-clamping and tumor removal followed by renorrhaphy. Temporary hilar-clamping reduces intraoperative bleeding, providing a clear surgical view, which facilitates precise tumor excision. However, hilar-clamping-induced renal ischemia and the subsequent reperfusion injury impair postoperative RF, critically depending on the ischemic time.^3^ Renorrhaphy addresses any necessary repair of the collecting system and cessation of renal bleeding but may contribute to renovascular complications, such as artery pseudoaneurysm and arteriovenous fistula.^4^

Recent improvements in the coagulation function of surgical energy devices such as radiofrequency^5^ and ultrasonic devices,^6^ microwave probes,^7,8^ and use of hemostatic materials^9–11^ have enabled renal bleeding control at the resected bed without renorrhaphy in clinical on- and off-clamp PNs. In on-clamp LPN, the “sutureless” approach using electrocoagulation and/or hemostatic materials reduced ischemic time, operative time, and blood loss, with oncologic control and a complication rate similar to that of renorrhaphy.^12^ Moreover, in off-clamp RPN, the surgical and functional outcomes of sutureless procedures using electrocoagulation without hemostatic materials are not inferior to those of renorrhaphy.^13^ These sutureless approaches are particularly beneficial in minimally invasive PNs, wherein workspace is limited,^14^ and renorrhaphy often requires sufficient dexterity, such as LPN and RPN. However, the available hemostatic methods limit the targets to small and superficial renal tumors.^12^

We previously proposed novel sutureless OPN^15^ and LPN^16^ techniques that allow for large tumor resections using only microwave scissors (MWS).^17^ Using these techniques, surgeons could conveniently carry out renal resection and adequately control renal bleeding without renorrhaphy because MWS can leverage the excellent heating performance of microwaves to seal and coagulate renal tissue or vessels and then mechanically cut them based on operator-dependent timing.^15,16^ In a porcine model, we demonstrated the feasibility of the off-clamp microwave scissors-based sutureless laparoscopic partial nephrectomy (MSLPN)^16^ technique, which has a short operative time and reduced blood loss. However, in case large vessels are inadvertently cut before proper coagulation and sealing are achieved, stopping the bleeding would be difficult even when using microwave energy. Therefore, the use of MWS during off-clamp sutureless LPN may require specific operator skills, or its use might be limited to certain clinical scenarios.

To overcome these limitations, we proposed the use of MSLPN with short-time hilar-clamping to minimize the risk of bleeding but still enhance operative outcomes. This proposal was supported by several reports indicating that the effects of limited renal ischemia on RF are negligible.^18–20^ In this study, we used a porcine model to compare the benefits and safety of on-clamp MSLPN with those of conventional open partial nephrectomy (cOPN).

## Methods

### Microwave Scissors

The MWS (Acrosurg Revo S; Nikkiso, Tokyo, Japan) are a scissors-shaped surgical device comprising two blades connected to the inner and outer conductors of a microwave-transmitted coaxial cable, which is used by connecting it to a 2.45-GHz frequency microwave generator. The MWS can seal and coagulate tissues by emitting microwaves into tissues contacted by both scissor blades.^17^ As we described previously,^16^ because the sealing and coagulation time and the cutting timing are arbitrarily adjustable, the MWS can be used flexibly and adaptively as cold scissors, as scissors for cutting with seamless sealing similar to radiofrequency and ultrasonic sealers, or as a simple coagulator without cutting. In this study, the generator’s power output was 60 W.

### Study Design

**Fig. 1:**
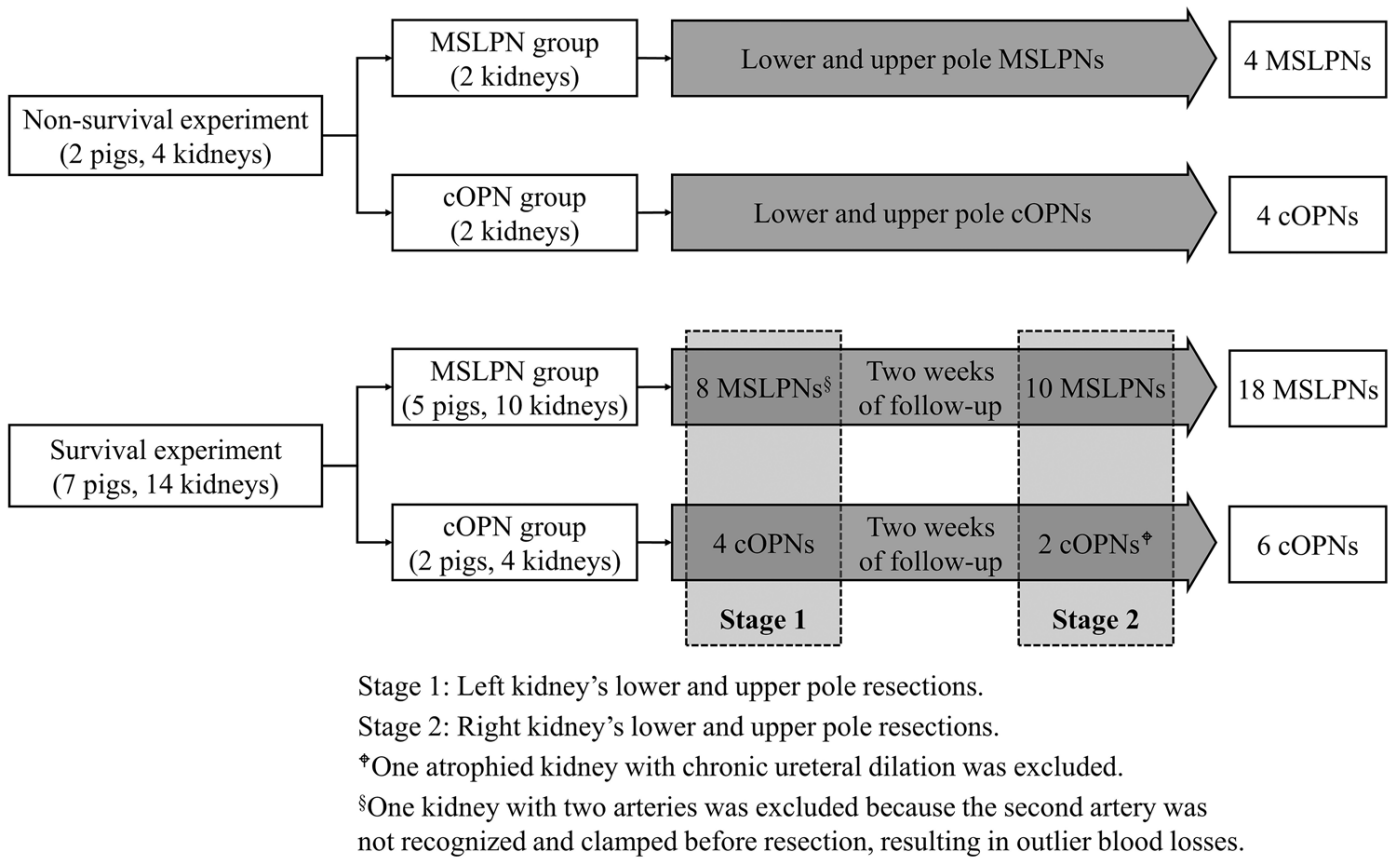
Study design and animal assignment. MSLPN, microwave scissors-based sutureless laparoscopic partial nephrectomy; cOPN, conventional open partial nephrectomy

This study used nine pigs with a median weight of 48 kg (range, 40–53 kg), which were raised in a pathogen-free environment at the Research Center for Animal Life Science of Shiga University of Medical Science. The study involved non-survival and survival experiments (Fig. 1). Two pigs were involved in the non-survival experiment, which was performed to try the MSLPN procedure as well as the MSLPN and cOPN perioperative outcome measurement processes. To resect the lower and upper poles, the left and right kidneys of each pig underwent MSLPN and cOPN, respectively.

The survival experiment involved seven pigs divided into MSLPN (*n* = 5) and cOPN (*n* = 2) groups to assess the postoperative complications. Each experiment included two stages. In the first stage, the lower and upper poles of the left kidney were sequentially resected. After 14 days of follow-up evaluation, the second stage involved operations to assess for postoperative complications and sequentially resect the right kidney’s lower and upper poles.

The pigs were euthanized immediately after the operations in the non-survival experiment or at the end of the second stage in the survival experiment. Kidney remnants were harvested for *ex vivo* pyelograms and histopathologic examination. Blood samples were collected before and after every procedure for hemoglobin (Hgb) and serum creatine (SrCre) level analyses using a Celltac α MEK-6550 machine (Nihon Kohden, Tokyo, Japan) and a multirotor I kidney profile panel on a VetScan VS2 machine (Abaxis, Union City, CA, USA), respectively.

### Procedures

**Fig. 2:**
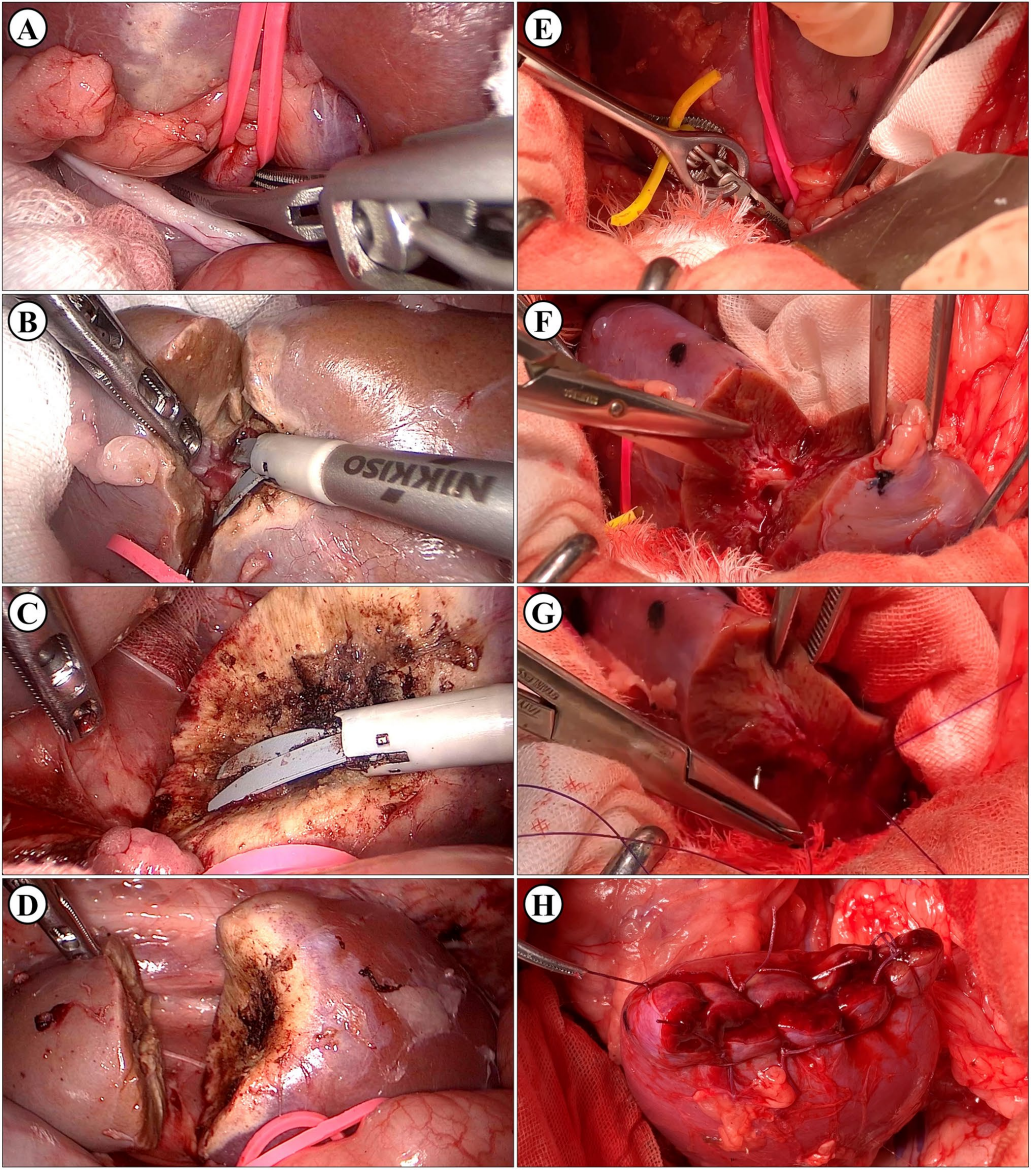
**A–D** Microwave scissors-based sutureless laparoscopic partial nephrectomy (MSLPN) and **E–H** conventional open partial nephrectomy (cOPN) of the kidney’s lower pole resections. **A, E** Renal arteries were temporarily clamped using a bulldog clamp. The kidney’s lower poles were resected using either **B** microwave scissors in MSLPN or **F** Metzenbaum scissors in cOPN. Resected beds were **C** coagulated using microwave scissors in MSLPN and **G** reconstructed via suturing in cOPN. After declamping, bleeding from renal remnants was completely controlled **D** without renorrhaphy in MSLPN or **H** through two-layer renorrhaphy in cOPN

The MSLPN procedure was performed using only MWS for renal resection and bleeding control (Fig. 2A–D). Transperitoneal laparoscopy was performed using four trocars, as we described previously.^16^ The kidney was exposed by detaching the posterior peritoneum and Gerota’s fascia. A vessel loop was used to isolate the renal artery. The resection line was marked using MWS by briefly scoring the kidney surface along the lower polar line.

The renal artery was temporarily clamped (Fig. 2A) using a laparoscopic bulldog clamp (H01AF; Heiwa Medical Instruments, Yamaguchi, Japan). The renal cortex was cut along the marking line, and the surgical margin was exposed by slight lifting of the resected tissue. The MWS were used to bite and seal the renal medullary parenchyma and vessels before they were mechanically cut (Fig. 2B). The renal calyces were exposed, sealed, and transected using the MWS. After specimen removal, the resected bed was coagulated using the MWS (Fig. 2C), and the renal artery was declamped. In cases of bleeding after declamping, the resected bed was recoagulated to consolidate hemostasis (Fig. 2D). The same kidney’s upper pole was then resected similarly.

With the pig in the lateral position, the cOPN procedure was performed via an open retroperitoneal approach, which involved temporary artery-clamping and renal resection followed by two-layer renorrhaphy (Fig. 2E–H). The procedure was initiated through flank-incision laparotomy below the 12th rib. The renal artery was isolated after opening of Gerota’s fascia and exposure of the kidney. After artery-clamping (Fig. 2E), the kidney’s lower/upper pole was resected using Metzenbaum scissors (Fig. 2F). Subsequently, renorrhaphy was performed in two layers. The renal medulla and the renal calyx openings were sutured (Fig. 2G) using a running suture (Monodiox 3-0; Alfresa Pharma, Osaka, Japan), whereas the renal cortex was reconstructed (Fig. 2H) using interrupting sutures (Opepolyx-N 0; Alfresa Pharma). If bleeding persisted after artery-declamping, additional suturing was performed.

### Procedural Exclusion

In the survival experiment, the final analysis excluded a kidney with two arteries in the MSLPN group because during renal resection, the second artery was unnoticed and left unclamped, resulting in outlier blood losses. During the operation, an atrophied kidney with chronic ureteral dilation was identified in the cOPN group and excluded (Fig. 1).

### Outcome Measurements

Data on specimen weight, specimen length, resection area (RA), procedure time (PT), ischemic time (IT [clamping time]), blood loss (BL), normal nephron loss (NNL), pre- and post-procedural Hgb and SrCre levels, and postoperative complications (including bleeding and extravasation of the remnant kidneys’ collecting systems during retrograde pyelograms) were recorded. The RA was calculated using the ellipsoid area formula, $S=\frac{\Pi }{4}{d}_{1}{d}_{2}$, where *d*_*1*_ and *d*_*2*_ indicate the resected specimen’s width and thickness, respectively. The PT was measured from the time of hilar-clamping to the time of complete renal bleeding control. The BL was determined by adding the suctioned blood volume to the blood volume estimated by subtracting the weight of preprocedural dry gauze from that of postprocedural blood-absorbed gauze.

In MSLPN and cOPN, NNL refers to the greatest depth of the thermal injury zone in the renal remnant and the suturing zone, respectively. Changes in Hgb and SrCre levels were determined by subtracting their preprocedural concentrations from their postprocedural concentrations. In the survival experiment, intra-abdominal conditions, including remnant kidney status, ascites, hematoma, and internal bleeding at the resection sites (if any), were recorded at the reoperation stage before the pigs were euthanized.

To assess how renal resection and bleeding control affect IT, the following parameters were recorded: resection time (RT), bleeding control time (BCT), bleeding control-related ischemic time (BCIT), and percentage of bleeding control-related ischemic time (%BCIT). The RT was measured from the time of hilar-clamping to the time of complete resected specimen removal. The BCT was measured from the time of resected specimen removal to complete cessation of renal bleeding using MWS (in MSLPN) or renorrhaphy (in cOPN). The BCIT was determined by subtracting the RT from the IT. The %BCIT was calculated by dividing the BCIT by the IT and then multiplying by 100.

### Statistical Analyses

Data were analyzed using SPSS Statistics for Windows, version 22.0 (IBM, Armonk, NY, USA). The non-parametric Mann–Whitney *U* and Fisher’s exact tests were used to compare the differences between two quantitative groups and two categorical groups, respectively. A *p* value lower than 0.05 indicated statistical significance.

## Results

Of the 22 MSLPNs and 10 cOPNs that we performed, 8 MSLPNs and 4 cOPNs in the survival experiments were followed up for 14 days after the operations. The perioperative outcomes of the two techniques are presented in Table 1. Specimen weight, specimen length, and RA did not differ significantly between the two groups. Compared with cOPN, MSLPN was associated with significantly lower PT (median, 9.2 vs 13.0 min;* p* = 0.026), IT (median, 5.9 vs 9.0 min; *p* < 0.001), BL (median, 14.4 vs 38.3 mL; *p* = 0.043), and NNL (median, 7.6 vs 9.4 mm; *p* = 0.004).

**Table 1:** The perioperative outcomes of MSLPN and cOPN in a porcine model

Parameter	MSLPN (*n* = 22)	cOPN (*n* = 10)	*p* Value^a^
SW (g), median (IQR)	20.7 (18.6–28.1)	19.0 (17.6–24.9)	0.515^b^
SL (mm), median (IQR)	30.2 (29.1–33.2)	29.6 (27.0–33.5)	0.166^b^
RA (cm^2^), median (IQR)	10.8 (9.7–12.6)	11.0 (9.7–13.7)	0.734^b^
PT (min), median (IQR)	9.2 (7.1–12.9)	13.0 (10.7–14.5)	**0.026**^b^
IT (min), median (IQR)	5.9 (5.3–6.8)	9.0 (8.1–9.6)	**< 0.001**^b^
BL (mL), median (IQR)	14.4 (9.4–28.5)	38.3 (12.0–74.9)	**0.043**^b^
NNL (mm), median (IQR)	7.6 (6.9–8.3)	9.4 (8.8–10.6)	**0.004**^b^
Hgb change^c^ (mg/dL), median (IQR)	− 0.8 (− 1.7–0.2)	− 0.9 (− 2.4–0.1)	0.667^b^
SrCre change^c^ (mg/dL), median (IQR)	0.0 (− 0.1–0.2)	0.2 (0.0–0.3)	0.269^b^
Postoperative bleeding, *n*	0	0	N/A
Extravasation on pyelogram, *n* (%)	12 (54.5)	3 (30.0)	0.265^b^
Pelvis stenosis on pyelogram, *n*	1	0	N/A

*MSLPN* Microwave scissors-based sutureless laparoscopic partial nephrectomy, *cOPN* Conventional open partial nephrectomy, *IQR* Interquartile range, *SW* Specimen weight, *SL* Specimen length, *RA* Resection area, *PT* Procedure time, *IT* Ischemic time, *BL* Blood loss, *NNL* Normal nephron loss, *Hgb* Hemoglobin, *SrCre* Serum creatinine, *N/A* Not applicable

^a^Bold font indicates statistically significant *p* value (*p* <0.05)

^b^Mann–Whitney *U* test and Fisher’s exact test

^c^Changes in Hgb and SrCre were determined by subtracting their preprocedural concentrations from corresponding postprocedural concentrations.

No cases of postoperative bleeding or acute kidney injury were observed during the experiments. We found no significant differences between the MSLPN and cOPN groups in the Hgb change (median, − 0.8 vs − 0.9 mg/dL; *p* = 0.667) or the SrCre change (median, 0.0 vs 0.2 mg/dL; *p* = 0.269).

**Fig. 3:**
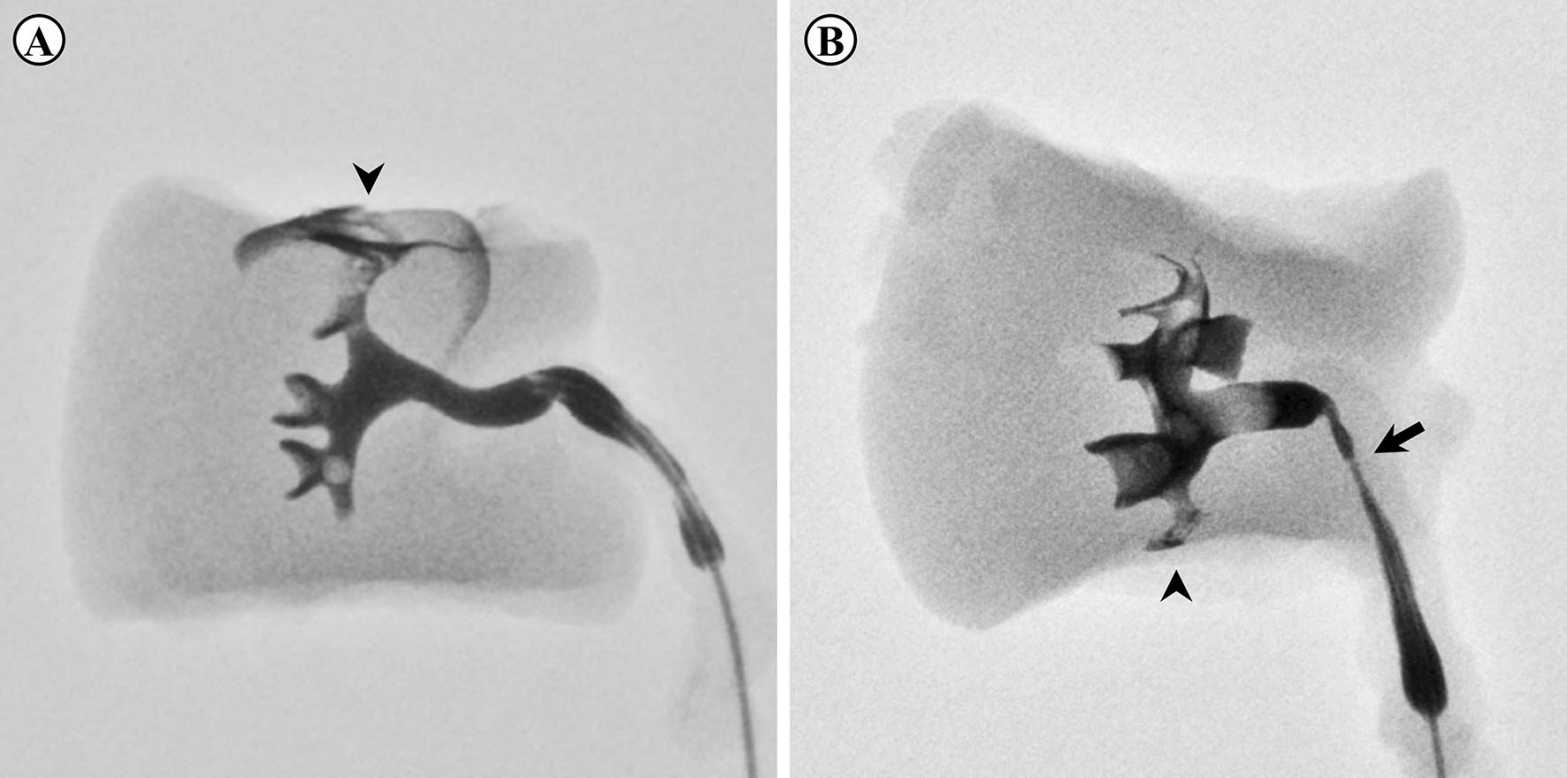
**A, B** Calyceal extravasation (*arrowheads*) and **B** renal pelvic stenosis (*arrow*) on *ex vivo* pyelograms after microwave scissors-based sutureless laparoscopic partial nephrectomy

All procedures involved lower or upper renal calyx transection. The extravasation rate was higher in the MSLPN group than in the cOPN group (54.5 % [*n* =12] vs 30.0 % [*n* = 3]), albeit without a statistically significant difference (*p* = 0.265). In the kidneys followed up for 14 days, extravasation was observed in five of the eight MSLPNs, but not in the four cOPNs. Pelvic stenosis was observed in one lower-pole MSLPN that involved deep resection near the renal hilum. The urinary extravasation and pelvic stenosis shown by *ex vivo* retrograde pyelograms after MSLPN are displayed in Fig. 3.

**Fig. 4:**
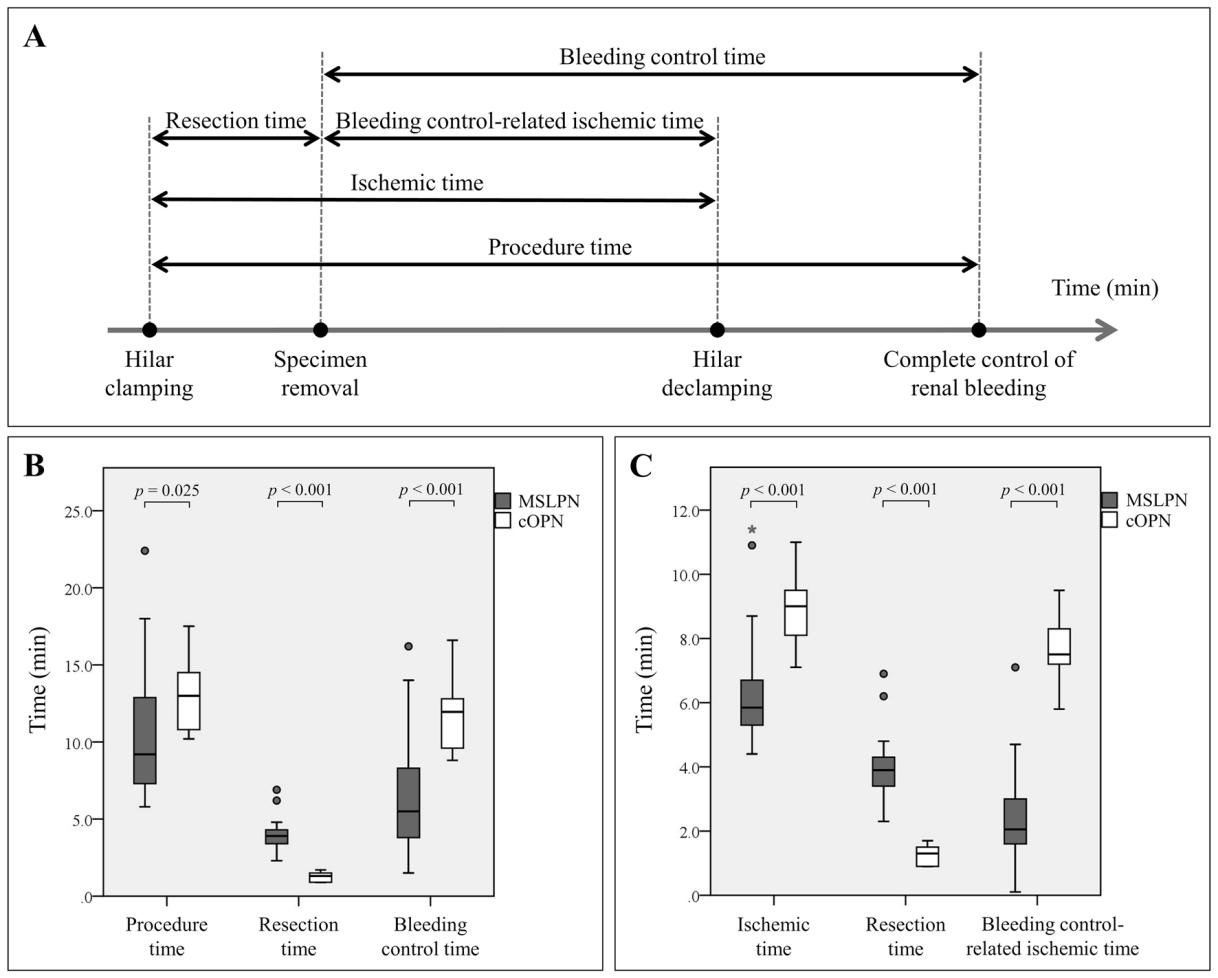
**A** Definition and **B**, **C** box-plot charts of **B** the procedure time, **C** the ischemic time, and their components in microwave scissors-based sutureless laparoscopic partial nephrectomy (MSLPN) and conventional open partial nephrectomy (cOPN). Although MSLPN **B**, **C** required more resection time, it markedly reduced **B** the bleeding control time and **C** the bleeding control-related ischemic time, thereby shortening **B** the procedure time and **C** the ischemic time compared with cOPN. Lower and upper box boundaries represent the first (Q1) and third (Q3) quartiles, respectively. The line inside the box indicates the median. The lower and upper error lines indicate the minimum and maximum values. Circles represent mild outliers (data points that lie more than one and a half times the interquartile range [IQR] below Q3 or above Q1). Asterisks indicate extreme outliers (data points that lie more than three times the IQR below Q1 or above Q3). Comparisons were made using the Mann–Whitney *U* test

**Fig. 5:**
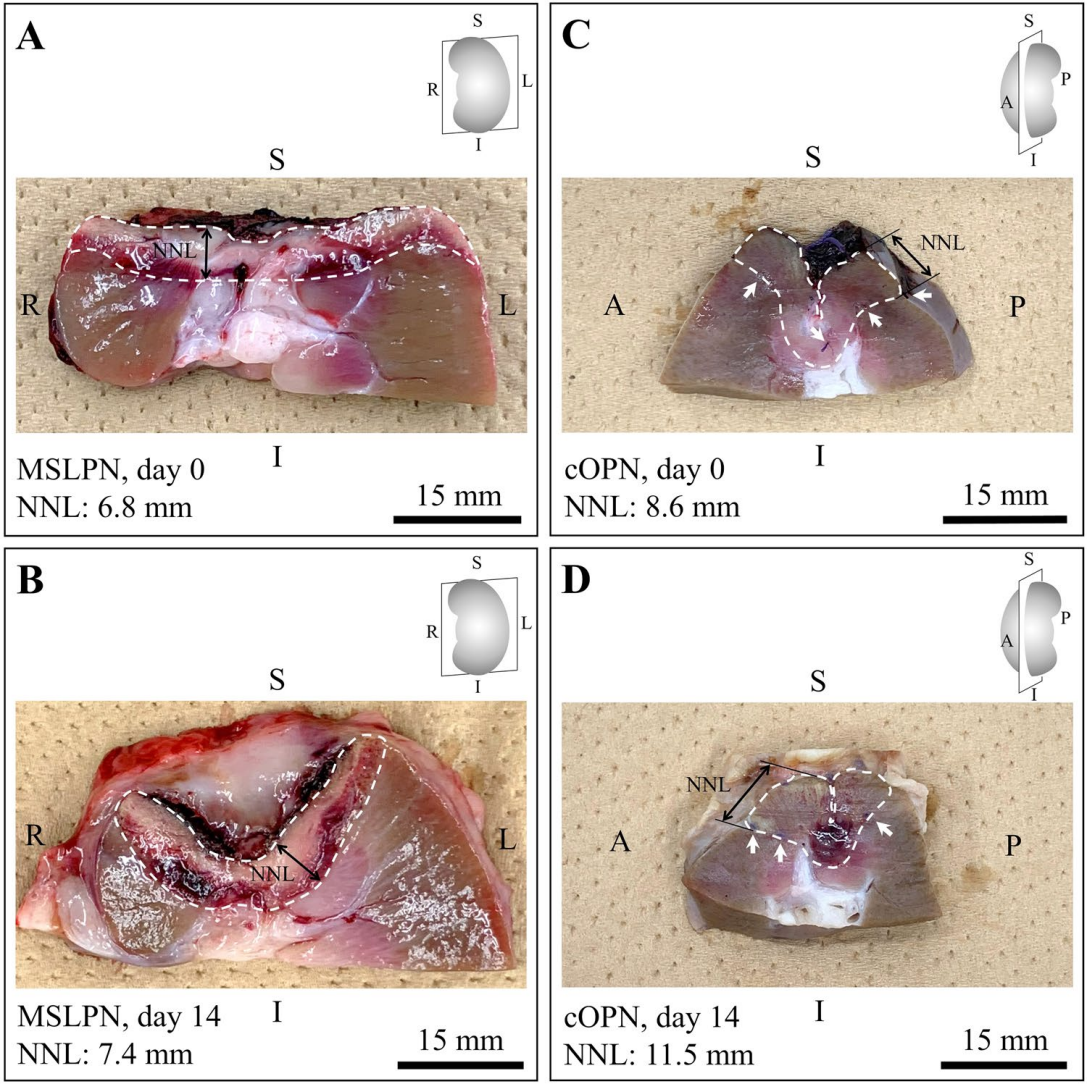
Renal remnants after MSLPN (**A** [day 0], **B** [day 14]) and cOPN (**C** [day 0], **D** [day 14]) sectioning on coronal and sagittal planes, respectively. The areas limited by the white dashed lines are the thermal injury zone induced by the microwave scissors in MSLPN and the suturing zone in cOPN. The white arrows indicate the suturing holes. MSLPN, microwave scissors-based sutureless laparoscopic partial nephrectomy; cOPN, conventional open partial nephrectomy; NNL, normal nephron loss; S, superior; I, inferior; R, right; L, left; A, anterior; P, posterior

The definition and box-plot charts comparing PT, IT, and their components across the two PN techniques are shown in Fig. 4. Although MSLPN required a longer RT than cOPN (median, 3.9 vs 1.3 min; *p* < 0.001), it markedly reduced the BCT (median, 5.5 vs 12.0 min; *p* < 0.001), BCIT (median, 2.1 vs 7.5 min; *p* < 0.001), and %BCIT (median, 40.6 % vs 86.0 %; *p* < 0.001), thereby shortening the PT and IT relative to cOPN. The remaining kidneys sectioned after MSLPN and cOPN are shown in Fig. 5. The histologic changes in renal tissue after MWS-based coagulation and renorrhaphy-induced devascularization were similar to those reported in our previous study.^15^

In MSLPN, we observed that tissue coagulation and sealing using MWS induced less surgical smoke, providing a clear laparoscopic view intraoperatively.

## Discussion

In this porcine study, the proposed MSLPN was associated with significantly shorter PT and IT as well as less BL and NNL than cOPN despite the well-known inferior dexterity of laparoscopic procedures compared with open procedures. The MWS enabled quicker renal resection and effective bleeding control at the resected bed in a short IT without renorrhaphy or hemostatic materials. However, urinary extravasation and pelvic stenosis occurred more frequently in the MSLPN group than in the cOPN group.

### Impacts of Renal Ischemia

For patients with a solitary kidney, off-clamp significantly reduces the risk of acute kidney injury and new-onset stage ≥3b chronic kidney disease compared with on-clamp PN,^21,22^ and an IT cutoff of 20 to 25 min may prevent RF decrease.^3,23^ This suggests that off-clamp PNs should be chosen when feasible for patients with a solitary kidney or a low baseline RF. For patients with two kidneys, regular baseline RF, and low-moderate complex tumors, two randomized controlled trials^19,20^ comparing off- and on-clamp RPN (mean IT, 19 min^19^; median IT, 15 min^20^) found no significant differences in decreases in absolute RF or surgically treated kidney’s split RF, measured postoperatively using scintigraphy at 3 months^19^ and 6 months.^20^ These studies demonstrated that on-clamp PNs with limited renal ischemia are acceptable for patients with two normal kidneys, and a limited IT shorter than 20 min may have only negligible effects on the RF of functioning kidneys.^18–20^

Although in conventional PNs, large^24^ and surgically complex tumors^25^ require a longer IT for tumor resection and renorrhaphy, renorrhaphy may be more technically demanding and therefore dominantly time-consuming compared with the tumor resection step. In this porcine study, renal resections were performed at the lower and upper polar line levels, mimicking the clinical removal of large renal tumors. The use of MWS enabled surgeons to quickly resect the renal parenchyma and immediately coagulate the resected bed, thereby stopping renal bleeding within a short IT (median IT, 5.9 min; interquartile range [IQR], 5.3–6.8 min) with less BL (median BL, 14.4 mL; IQR, 4.4–28.5 mL). Therefore, if MSLPN is applied to clinical scenarios, tumor resection and control of renal bleeding would be accomplished within a limited IT that supposedly affects postoperative RF only negligibly.

### Impacts of Renorrhaphy

In conventional PNs, double-layer suturing (inner and outer) is traditionally performed to repair the renal calyx if needed and reconstruct parenchymal defects after tumor resection, stopping renal bleeding.^26^ However, renal suturing may lead to renal dysfunction^27^ because of increased IT and loss of healthy parenchyma.^8–10,12^ Using computed tomography imaging to measure pre- and postoperative kidney volumes, Mir et al.^28^ showed that 83 % (IQR, 75–91 %) of functioning parenchyma was preserved and that preserved healthy parenchyma was the strongest predictor of saved RF. This indicates a 17 % loss in functioning parenchyma and that healthy parenchyma loss, including parenchyma excision with the tumor and devascularization during reconstruction, may play a key role in RF decrease after PN. Using a similar volumetric analysis and presuming that healthy parenchyma loss involves a 5-mm parenchyma rim from the tumor border and any radially located tissue, Takagi et al.^29^ still demonstrated an additional 7 % decrease in parenchymal volume after PN. These findings indicate that the depth of parenchyma loss might be greater than presumed.

Currently, tumors can be removed through enucleation or enucleoresection,^30^ which involves a thin rim along the tumor pseudo-capsule to minimize parenchyma excision with the tumor. Therefore, healthy parenchyma loss after PN may occur mainly through parenchyma devascularization. In this study, suture-based bleeding control was associated with a 9-mm depth of devascularized parenchyma loss, raising concerns about the potentially detrimental effects of parenchyma reconstruction on RF preservation. Moreover, intracorporeal suturing, performed in the race against IT and thus requiring dexterous surgical instrument manipulation, is technically challenging in minimally invasive PNs like LPN and RPN,^31^ with renal vessels occasionally damaged by the suture needles, resulting in artery pseudoaneurysms or arteriovenous fistula.^4^

To achieve hemostasis, sutureless PN uses energy devices^5–10^ and hemostatic materials.^9–11^ Sutureless PN has the advantages of being simple, less technically challenging, and easy to learn and apply to minimally invasive PNs. Several studies^8–10,12^ comparing sutureless and conventional PN have reported significant advantages in terms of IT, operative time, and decreased RF favoring sutureless PN. However, sutureless PN requires reliable hemostatic methods in kidneys because failure to successfully control renal bleeding using energy devices may require renal suturing, which would be more difficult because of thermal coagulation-induced tissue fragility. Therefore, sutureless PNs are currently limited to selected patients with small and superficial renal tumors. Although it is reported that bipolar radiofrequency and ultrasonic sealers can seal vessels up to 7 mm in size^32^ and that they can be used for sutureless PNs,^5,6^ these devices require tissue grasping with the sealer’s jaws, as well as pressure application, coagulation, and then cutting using a built-in blade or the cavitation effect. These maneuvers are time-consuming, and when solid organs such as kidneys are resected, the tissue may be crushed before proper coagulation is achieved.

Considering these challenges, for renal parenchyma resection, the scissors-shaped tip of MWS is more advantageous than jaw-shaped tip devices. Additionally, MWS-based coagulation has excellent hemostasis capability in kidneys,^7,15,16^ but as shown in this study, it results in less NNL than conventional renorrhaphy. As discussed in our previous study,^15^ this may result from the instantaneous tissue coagulation and fixation effects^33^ induced by rapid and direct microwave heating. However, our data showed that if renal resection involves calyx transection, solely sealing the calyx using MWS is not sufficient to prevent urine leakage.

### Advantages, Disadvantages, and Clinical Viewpoints on MSLPN

We noted that the scissors-shaped tip of the MWS enables surgeons to use their quick renal excision skills, similar to those achieved with conventional laparoscopic scissor instruments. Furthermore, the interblade microwave coagulation function provides a smokeless tissue-coagulating environment. This ensures renal bleeding control while maintaining a clear surgical margin within a short IT, thereby reducing the intracorporeal laparoscopic suturing burden required for the reconstruction of parenchymal defects to stop bleeding. Moreover, MSLPN reduced the IT, NNL, and their associated risks of postoperative RF deterioration compared with cOPN. Taken together, our data highlight MSLPN as a novel minimally invasive surgical treatment for improving operative outcomes of patients with localized renal tumors.

However, MSLPN may be associated with an increased rate of urinary complications. In this study, all the MSLPN and cOPN procedures involved calyceal transection to test the calyceal-sealing function of MWS and to compare it with conventional renorrhaphy. Urinary extravasation occurred more frequently in MSLPN (54.5 %) than in cOPN (30.0 %), which is a concern about the MSLPN technique. Because microwave coagulation timing and mechanical cutting can be adjusted arbitrarily, using MWS, surgeons can resect renal tissues flexibly and control bleeding adaptively while confirming the tissue conditions visually. However, the possibility of early cutting before the renal calyces are completely sealed may result in urinary extravasation on pyelograms. Urine leakage is the most commonly reported complication, which occurs in about 7 % (range, 1.4–17.4 %) of all PN cases.^34^ However, the urine leakage rate may be higher in PN cases, wherein the collecting system is violated, reaching 44 %,^35^ especially if a major reconstruction of the collecting system is required. Tumors with direct pelvicalyceal contact are an independent risk factor,^36^ leading to a threefold increase in urine leakage after PN. Additionally, in the MSLPN group, pyelograms showed a renal pelvic stenosis case caused by thermal injury after a deep lower pole resection involving the renal hilum.

These advantages and disadvantages indicate that if MSLPN is used, patients with tumors located away from renal calyces would benefit the most from improved operative outcomes while avoiding calyceal violation. In cases with tumors close to renal calyces, MSLPN could be cautiously used, and if calyceal violations occur during tumor resections, additional repair of the renal calyces via an inner suturing after bleeding control should be performed to prevent urine leakage. Although this calyceal-suturing step may not require more IT, surgeons should pay attention to tissue fragility after thermal coagulation and the possibility of transecting medullary vessels with the suture needle. For tumors near the renal hilum, neither MSLPN nor sutureless PNs using energy devices should be used to avoid thermal injury of the renal pelvis.

This study had several limitations. First, the sample was small. Pig kidneys are not as well vascularized as human kidneys. Second, although renal resections at the lower and upper polar line levels were used to evaluate the control of bleeding from large medullary vessels, the polar resection of healthy, tumor-free kidneys did not mimic the tumor location variety and their complexity in clinical scenarios. Finally, because MSLPN was not compared with sutureless techniques that do not involve MWS, the advantages of MWS over other hemostatic methods in sutureless PN remain uncertain.

Although LPN numbers are markedly decreasing and the minimally invasive PN trend is shifting from LPN to RPN,^37^ for centers without robots, LPN remains a viable, cost-effective option. The MSLPN technique, with its less technically demanding procedures, can offer an affordable minimally invasive surgical treatment method for patients with localized renal tumors. Moreover, if surgical robots are equipped with MWS, the resulting “MWS-based sutureless RPN” could provide a dexterous MWS manipulation with enhanced procedural execution.

In conclusion, we propose “on-clamp MSLPN,” a novel on-clamp sutureless LPN technique that uses only MWS for tumor resection and bleeding control. Our data show that in minimally invasive PNs, on-clamp MSLPN improves intraoperative outcomes while reducing the technical demands. These findings indicate that for selected patients with non-hilar localized renal tumors, on-clamp MSLPN can be used. However, its use on tumors near renal calyces should be cautious, and the calyces, if violated, should be sutured to prevent urine leakage.
